# Emergence of Genetic Diversity and Multi-Drug Resistant *Campylobacter jejuni* From Wild Birds in Beijing, China

**DOI:** 10.3389/fmicb.2019.02433

**Published:** 2019-10-29

**Authors:** Juan Du, Jing Luo, Jingjing Huang, Chengmin Wang, Meng Li, Bojun Wang, Bo Wang, Han Chang, Jianwei Ji, Keya Sen, Hongxuan He

**Affiliations:** ^1^National Research Center for Wildlife Borne Diseases, Institute of Zoology, Chinese Academy of Sciences, Beijing, China; ^2^College of Life Sciences, University of the Chinese Academy of Sciences, Beijing, China; ^3^Guangdong Key Laboratory of Animal Conservation and Resource Utilization, Guangdong Public, Laboratory of Wild Animal Conservation and Utilization, Guangdong Institute of Applied Biological Resources, Guangzhou, China; ^4^Beijing Wildlife Rescue Center, Beijing Municipal Bureau of Landscape and Forestry, Beijing, China; ^5^Division of Biological Sciences, Science, Technology, Engineering and Mathematics, University of Washington, Bothell, WA, United States

**Keywords:** emergence, *Campylobacter jejuni*, wild birds, MLST, multi-resistance, CDT gene cluster

## Abstract

*Campylobacter jejuni* (*C. jejuni*) is considered as an opportunistic zoonotic pathogen that may cause gastroenteritis in humans and other animals. Wild birds may be as potential vectors of *C. jejuni* around urban and suburban areas. Here, 520 samples were collected from 33 wild bird species in urban and suburban areas, Beijing. In total 57 *C. jejuni* were isolated from seven species. It was found that Nineteen (33.33%, 19/57) isolates were resistant to at least one of 11 antibiotics, especially streptomycin (36.84%) and four isolates resistant to all. Nineteen (33.33%, 19/57) isolates were multi-drug resistance. Multilocus sequence typing (MLST) analysis of the isolates showed that 36 different sequence types (STs) belonged to four Clonal complexes and unassigned. Twenty STs (55.56%) and six alleles among them were first detected. Virulence genes including *flaA*, *cadF*, and the cytolethal distending toxin (CDT) gene cluster, were detected in all isolates, but truncated *cdt* gene clusters only detected in the isolates from the crow, daurian jackdaw and silver pheasant. In conclusion, it was the first detection of *C. jejuni* involved truncated *cdt* gene clusters from the silver pheasant. These wild birds around urban and suburban areas may pose potential public health problems as reservoir vectors of *C. jejuni*.

## Introduction

*Campylobacter jejuni* is a gram-negative spiral rod bacterium that causes gastroenteritis in humans and other animals. In 2016, a total of 246,307 confirmed cases of human campylobacteriosis were reported in the European Union (EU), representing almost 70% of all the reported human cases of zoonoses (European Food Safety Authority (EFSA) and European Centre for Disease Prevention and Control (ECDC), 2017). There was a significantly increasing trend over the period 2008–2016. Compared with European countries, reports on campylobacteriosis in Asian countries, including China are limited, and the campylobacteriosis prevalence in humans is generally low (Wang et al., 2015).

The clinical presentation of campylobacteriosis includes watery or bloody diarrhea lasting for a median duration of 6 days, with 80% of patients having cramps and fever (Friedman et al., 2004). In some cases, the infection can lead to extra-intestinal complications and severe autoimmune disorders, such as pancreatitis, cholecystitis, obstructive hepatitis, Guillain-Barré syndrome (GBS), and Miller Fisher syndrome. Generally, the patients with campylobacteriosis are self-limited and disappear after 1 week without any specific treatment. However, in some relatively severe cases, antimicrobial chemotherapy is required. Macrolides and quinolones are commonly used as first-line therapies, and tetracycline, doxycycline, and chloramphenicol are alternative drugs (Ma et al., 2017).

Resistant strains and multi-drug resistance (MDR) strains are increasingly reported in humans and animals which may be induced by increasing use of antibiotics in humans, domestic animals and poultry (Franiczek et al., 2012; Karaiskos and Giamarellou, 2014; Alsan et al., 2015). *C. jejuni* has a high capacity to transfer genetic elements that lead to the combination of different strains. This characteristic may allow of *C. jejuni* transfer antibiotic resistance genes easily (de Boer et al., 2002; Wilson et al., 2003; Avrain et al., 2004), The abuse of antibiotics could increase the selection pressure and decrease the effectiveness of antibiotics further (Klena et al., 2004; Laxminarayan et al., 2013).

In *Campylobacter*, mutations in the 23S rRNA genes shown to contribute to macrolide resistance (Payot et al., 2006). *C. jejuni* resistance to tetracycline is usually associated with the *tet(O)* gene, which is carried on transmissible plasmids or located chromosomally. Ribosomal protein – Tet(O), which encoded by *tet(O)* gene can confer the resistance by displacing tetracycline from its primary binding site on the ribosome (Connell et al., 2003; Gibreel et al., 2004b). *C. jejuni* resistance to fluoroquinolones is mainly associated with mutations in the DNA gyrase gene (*gyrA*) (Smith and Fratamico, 2010). Accordingly, it is necessary to monitor the antibiotic resistance and research the antibiotic resistance mechanism of *C. jejuni* in wildlife.

The process of infection involved in adhesion, colonization, invasion and toxin production, especially, *flaA* genes, *cadF* genes, and *cdt* gene clusters are necessary for cell pathology and virulence in humans (Wei et al., 2018). These three virulence genes are frequently researched in isolates from various hosts, especially humans and poultry. However, little is known about these virulence genes in wild bird isolates (Wei et al., 2018).

The consumption of feces-contaminated raw or undercooked poultry has been identified as an important transmission vehicle for human campylobacteriosis (Wei et al., 2018). However, there is evidence that non-food-borne exposure of *C. jejuni* may contribute to the burden of illness as well. Thus contamination of the environment by domestic and wild birds feces may constitute an additional risk for human infection via environmental water or direct contact with them (French et al., 2009). Some studies suggested that *C. jejuni* is a commensal microorganism in the intestine of many wild and domestic animals, particularly avian species, and they can be natural reservoirs of *C. jejuni* (Oravcova et al., 2014). Furthermore, migratory birds may play a direct or indirect role in the zoonotic transmission of *Campylobacter* through the foods with fecal contamination (Kapperud et al., 2003; Gardner et al., 2012). *C. jejuni* has been isolated from wild birds such as pigeons, crows, geese, ducks, gulls, and cranes (Broman et al., 2002; Chen et al., 2011). Isolates from black-headed gulls (*Chroicocephalus ridibundus*), Sandhill cranes (*Grus canadensis*), and European starlings (*Sturnus vulgaris*) have been implicated in human disease (Palmer et al., 1983; Broman et al., 2002, 2004; Waldenstrom et al., 2002; Pearson et al., 2007; Karaiskos and Giamarellou, 2014).

Although *C. jejuni* is the main cause of bacterial diarrhea, there are still limited data on *C. jejuni* in China, especially for a wide range of free-living and migrating birds in various parts of cities, and the threat to public health cannot be ignored. Here, we investigated the prevalence, genetic diversity (Multilocus sequence typing), antimicrobial resistance patterns and virulence genes (*flaA* genes, *cadF* genes, and *cdt* gene cluster) of Campylobacter in wild birds in Beijing, China.

## Results

### The Prevalence of *Campylobacter jejuni*

**FIGURE 1:**
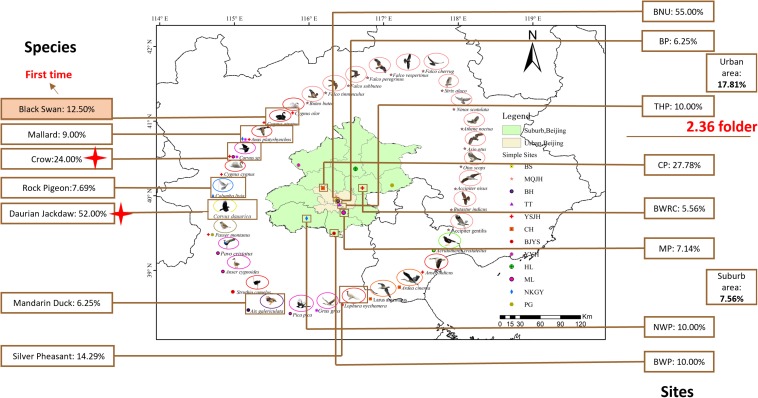
Samples collect sites and species in Beijing, China. Green represent Suburb area, Beijing; fleshcolor represent Urban area, Beijing. Images around the map of Beijing represent 33 species and the small icons represent 11 different sites. The data in the box represent the rate of the positive *C. jejuni* samples from different species and sites. Red tetragon represent crows and daurian jackdaws of family *Corvids* have significantly high positive rate than other species (*P* < 0.007). BNU, Beijing Normal University; BP, Beihai Park; THP, Temple of Heaven Park; CP, Cuihu Park; BWRC, Beijng Wildlife rescue center; BWP, Beijing Wildlife Park; MP, Milu Park; NWP, Niukouyu Wetland Park; BRRC, Beijing Raptor rescue center; WDL, Wild Duck Lake; HL, Hongluo Lake; XR, Xiyu Reservoir.

In total, 520 samples were collected from 33 species and 12 sites during the 4 months in Beijing, China (Figure 1). *C. jejuni* was isolated from 57 samples (10.96%, 57/520) including seven species. In the positive *C. jejuni* samples, 24.19% (15/62) were from crow (*Corvus* sp.), 51.67% (31/60) were from daurian jackdaw (*Corvus dauurica*), 14.29% (1/7) were from silver pheasant (*Lophura nycthemera*), 8.57% (6/70) were from mallard (*Anas platyrhynchos*), 6.25% (1/16) were from mandarin duck (*Aix galericulata*), 12.5% (1/8) were from black swan (*Cygnus atratus*) and 7.69% (2/26) were from rock pigeon (*Columba livia*). Crows (*Corvus* sp.) and Daurian jackdaws (*Corvus dauurica*) had significantly higher positive rates than other species (*P <* 0.007). Mallard (*Anas platyrhynchos*), mandarin duck (*Aix galericulata*) and black swan (*Cygnus atratus*) showed a relatively low positive rate between 6.25 and 12.5%. Silver Pheasant (*Lophura nycthemera*), family *Phasianidae* showed a similar positive rate as chicken, which is one of the most important reservoirs of *C. jejuni*. Rock pigeon (*Columba livia*), had relatively lower positive rate than others. This is the first time that *C. jejuni* has been isolated from black swan (*Cygnus atratus*) (Table 1).

**TABLE 1:** Prevalence of *Campylobacter jejuni* isolates from different species.

**Family**	**Species**	**Number**	**Isolation rate (%)**	**Family**	**Species**	**Number**	**Isolation rate (%)**
Accipitridae	European Sparrowhawk (*Accipiter nisus*)	3	0	Falconidae	Eurasian Kestrel (*Falco tinnunculus*)	19	0
Accipitridae	Gray-faced Buzzard (*Butastur indicus*)	1	0	Falconidae	Eurasian Hobby (*Falco subbuteo*)	7	0
Accipitridae	Eurasian Goshawk (*Accipiter gentilis*)	1	0	Falconidae	Peregrine Falcon (*Falco peregrinus*)	2	0
Accipitridae	Common Buzzard (*Buteo buteo*)	7	0	Falconidae	Red feet falcon (*Falco amurensis)*	2	0
Anatidae	Mallard (*Anas platyrhynchos*)	70	9%	Gruidae	Crane (*Grus grus*)	22	0
Anatidae	Mandarin duck (*Aix galericulata*)	16	6.25%	Laridae	Herring Gull (*Larus argentatus*)	46	0
Anatidae	Black Swan (*Cygnus atratus*)	8	12.50%	Paridae	Sparrow (*Passer montanus*)	50	0
Anatidae	Swan Goose (*Anser cygnoides*)	10	0	Phasianidae	Silver Pheasant (*Lophura nycthemera*)	7	14.29%
Anatidae	Bar-headed Goose (*Anser indicus*)	7	0	Phasianidae	Common Peafowl (*Pavo cristatus*)	31	0
Anatidae	Mute Swan (*Cygnus olor*)	4	0	Strigidae	European Scops Owl (*Otus scops*)	12	0
Anatidae	Swan (*Cygnus* sp.)	3	0	Strigidae	Long-eared Owl (*Asio otus*)	2	0
Ardeidae	Gray Heron (*Ardea cinerea*)	23	0	Strigidae	Little Owl (*Athene noctua*)	1	0
Columbidae	Rock Pigeon (*Columba livia*)	26	7.69%	Strigidae	Eagle owl (*Ninox scutulata*)	2	0
Corvidae	Crow (*Corvus* sp.)	62	24%	Strigidae	Tawny Owl (*Strix aluco*)	1	0
Corvidae	Daurian Jackdaw (*Corvus dauurica*)	60	52%	Struthionidae	Common Ostrich (*Struthio camelus*)	3	0
Corvidae	Magpie (*Pica pica*)	1	0	Sturnidae	Crested Myna (*Acridotheres cristatellus*)	7	0
Falconidae	Saker falcon (*Falco cherrug*)	4	0				

The 12 collection sites were divided into two groups, including urban area and suburban area. Totally, all the positive samples of *C. jejuni* were from 8 sites, including Beijing Normal University (BNU) (55.00%), Beihai Park (BP) (6.25%), Temple of Heaven Park (THP) (10.00%), Cuihu Park (CP) (27.78%), Beijing Wildlife Rescue Center (BWRC) (5.56%), Beijing Wildlife Park (BWP) (10.00%), Milu Park (MP) (7.14%), and Niukouyu Wetland Park (NWP) (10.00%). The highest was BNU (55%) which located in an urban area, and the lowest was BWRC (5.46%) which located in the suburb area. The average positive rate in urban areas (17.81%) was 2.36 times higher than in suburb areas (7.66%) (Table 2).

**TABLE 2:** Prevalence of *Campylobacter jejuni* isolates from different sites.

**Location**	**Site**	**Sample number**	**Isolation rate (%)**	**Average isolation rate (%)**
	BNU	20	55.00	
Urban area	BRRC	64	0.00	17.81
	BP	16	6.25	
	THP	40	10.00	
	BWRC	72	5.56	
	CP	90	27.78	
	BWP	10	10.00	
Suburb area	WDL	22	0.00	7.56
	HL	7	0.00	
	MP	98	7.14	
	NWP	40	10.00	
	XR	41	0.00	

*^1^BNU, Beijing Normal University; BP, Beihai Park; THP, Temple of Heaven Park; CP, Cuihu Park; BWRC, Beijng Wildlife rescue center; BWP, Beijing Wildlife Park; MP, Milu Park; NWP, Niukouyu Wetland Park; BRRC, Beijing Raptor rescue center; WDL, Wild Duck Lake; HL, Hongluo Lake; XR, Xiyu Reservoir.*

### Multilocus Sequence Typing for *Campylobacter jejuni*

To discover the genetic diversity of *C. jejuni* in wild birds and explore the role of wild birds in disease transmission, the genotype of *C. jejuni* isolates from wild birds were tested by multilocus sequence typing (MLST). MLST, which uses seven genes to build a classification system, is a common way to reveal genetic diversity. Fifty-seven isolates were divided into 36 different sequence types (STs) that clustered into four clonal complexes (CCs) and unassigned.

The same bird species could carry a variety of STs from different individuals (Table 3). Twenty-three STs were identified from 31 *C. jejuni* isolates in daurian jackdaws, and 7 STs from 15 isolates in crows. Overall, 52.6% of novel STs were first discovered in the present study (Table 3), especially more than 10 novel STs were from one or more new allelic genes, and six novel alleles were found (aspA484, aspA485, glnA673, glyA754, tkt718, tkt719) (Supplementary Table S1). Moreover, 84.5% (49 strains) of the STs did not belong to any clonal complex (CC). Three STs (ST-692, ST-991, ST-9191) belonged to ST-692 clonal complex and ST-52, ST-952, and ST-1275 complex comprised three STs, respectively (Table 3).

**TABLE 3:** Clonal complex (CC) and sequence type (ST) distribution of *Campylobacter* in wild bird species.

**CC**	**ST**	**Number**	**Avian species**	**Site**
			**(No. of isolates)**	
52	52	1	Black Swan (1)	BERC
692	692	1	Mandarin Duck (1)	BP
	991	2	Mallard (2)	NWP
	**9191**	1	Mallard (1)	BWSC
952	**9176**	2	Crow (2)	THP
1275	1540	1	Daurian Jackdaw (1)	CP
U	448	4	Daurian Jackdaw (4)	MP
	951	1	Daurian Jackdaw (1)	CP
	953	2	Daurian Jackdaw (2)	CP
	995	1	Mallard (1)	CP
	999	1	Daurian Jackdaw (1)	CP
	2367	2	Pigeon (2)	NWP
	3938	1	Daurian Jackdaw (1)	CP
	4069	1	Daurian Jackdaw (1)	CP
	4382	2	Crow (2)	BNU
	4571	1	Crow (1)	BNU
	6168	1	Daurian Jackdaw (1)	CP
	7805	1	Daurian Jackdaw (1)	CP
	**9175**	4	Crow (4)	BNU
	**9177**	2	Daurian Jackdaw (2)	MP/CP
	**9178**	2	Daurian Jackdaw (1)	MP
	**9179**	1	Daurian Jackdaw (1)	CP
	**9180**	2	Daurian Jackdaw (2)	CP
	**9181**	1	Daurian Jackdaw (2)	CP
	**9182**	1	Daurian Jackdaw (2)	CP
	**9183**	2	Daurian Jackdaw (2)	CP
	**9184**	1	Daurian Jackdaw (2)	CP
	**9185**	2	Daurian Jackdaw (2)	CP
	**9186**	1	Daurian Jackdaw (2)	CP
	**9190**	2	Mallard (2)	BWRC
	**9192**	2	Crow (2)	BNU
	**9194**	1	Daurian Jackdaw (2)	CP
	**9195**	1	Daurian Jackdaw (3)	CP
	**9196**	2	Crow (2)	THP/BNU
	**9197**	2	Crow (3)	THP
	**9222**	2	Daurian Jackdaw (1)	CP
			Silver Pheasant (1)	BWP

*^1^U, Unassigned clonal complex. ^2^Novel STs are indicated in bold. ^3^In parentheses, the number of isolates from each bird. ^3^BNU, Beijing Normal University; BP, Beihai Park; THP, Temple of Heaven Park; CP, Cuihu Park; BWRC, Beijng Wildlife rescue center; BWP, Beijing Wildlife Park; MP, Milu Park; NWP, Niukouyu Wetland Park; BRRC, Beijing Raptor rescue center; WDL, Wild Duck Lake; HL, Hongluo Lake; XR, Xiyu Reservoir.*

### Phylogenetic Analysis of *Campylobacter jejuni* Strains

The minimum spanning tree for 57 C. *jejuni* isolates and other reference isolates from the PubMLST database was constructed (Supplementary Figure S1). The genetic diversity of the C. *jejuni* isolates showed that nine STs (ST-995, ST-991, ST-2367, ST-3938, ST692, ST-52, ST1540, ST448, ST-951, ST4069) were found in wild birds and human, 5 STs (ST-995, ST-991, ST-2367, ST-52, ST692, ST1540) in wild birds, chicken and humans, 2 STs (ST-448 and ST-995) in monkeys, wild birds and humans, and 2 STs (ST-52 and ST-995) in 4 hosts (wild birds, humans, chicken, dogs/monkeys). Researches previously reported showed that humans and other animals have the same predominant STs, which was suggested that these animals are important reservoirs of human domestically acquired infections (Olkkola et al., 2016). In this study, the isolates from black swan, mandarin duck, mallard, daurian jackdaw, and rock pigeon were found in humans (Table 4). Therefore these results above indicated that the *C. jejuni* could transmit between different species (Tables 3, 4 and Supplementary Figure S1).

**TABLE 4:** Sequence types (STs) and their association certain species and area.

**STs**	**Species**	**Area**	**STs**	**Species**	**Area**
52	Human stool	United Kingdom/United States/Brazil/Israel/Botswana/	995	Human stool	Sweden
		Germany/Sweden/Luxembourg/		Wild birds	Sweden/Finland
		Switzerland/Canada/Japan/		Chicken	Canada/Sweden/United Kingdom
		The Netherlands/Australia/Greece		Duck	New Zealand
	Sheep	United Kingdom		**Mallard**	**China**
	Chicken	United Kingdom/United States/New Zealand/	3938	Human stool	Sweden
		Spain/Luxembourg/		**Daurian Jackdaw**	**China**
		Senegal/Switzerland/Uruguay	4069	Human stool	Canada
	Cattle	United Kingdom		**Daurian Jackdaw**	**China**
	Xiangjiang River	**China**	953	Wild birds	United Kingdom
	Monkey	**China**		**Daurian Jackdaw**	**China**
	**Black Swan**	**China**	999	Starling	United Kingdom
692	Human stool	United Kingdom		Wild birds	United Kingdom
	Goose	United Kingdom		**Daurian Jackdaw**	**China**
	Chicken	The Netherlands/	4382	Wild birds	Canada
		Luxembourg/China		**Crow**	**China**
	Wild birds	Sweden	4571	/	Finland
	Environmental waters	United States/New Zealand		**Crow**	**China**
	**Mandarin Duck**	**China**	6168	Environmental waters	Luxembourg
991	Human stool	United Kingdom/Luxembourg/		**Daurian Jackdaw**	**China**
		New Zealand/Germany	7805	Wild birds	Finland
	Environmental waters	Canada		**Daurian Jackdaw**	**China**
	Human stool	United Kingdom/United States	**9175**	**Crow**	**China**
	Wild birds	Sweden/New Zealand/Finland	**9176**	**Crow**	**China**
	Chicken	United Kingdom	**9177**	**Daurian Jackdaw**	**China**
	Sheep	New Zealand	**9178**	**Daurian Jackdaw**	**China**
	Environmental waters	Canada	**9179**	**Daurian Jackdaw**	**China**
	**Mallard**	**China**	**9180**	**Daurian Jackdaw**	**China**
1540	Human stool	United Kingdom	**9181**	**Daurian Jackdaw**	**China**
	Chicken	United Kingdom	**9182**	**Daurian Jackdaw**	**China**
	Environmental waters	Luxembourg	**9183**	**Daurian Jackdaw**	**China**
	Wild birds	United States/Japan	**9184**	**Daurian Jackdaw**	**China**
	**Daurian Jackdaw**	**China**	**9185**	**Daurian Jackdaw**	**China**
448	Human stool	United Kingdom/Switzerland/Sweden	**9186**	**Daurian Jackdaw**	**China**
	Wild birds	United Kingdom/Japan/United States	**9190**	**Mallard**	**China**
	Environmental waters	Canada/The Netherlands/France	**9191**	**Mallard**	**China**
	**Daurian Jackdaw**	**China**	**9192**	**Crow**	**China**
951	Human stool	United Kingdom	**9194**	**Daurian Jackdaw**	**China**
	Wild birds	United Kingdom	**9195**	**Daurian Jackdaw**	**China**
	**Daurian Jackdaw**	**China**	**9196**	**Crow**	**China**
2367	Human stool	United Kingdom	**9197**	**Crow**	**China**
	Chicken	Germany	**9222**	**Daurian Jackdaw/**	**China**
	**Pigeon**	**China**		**Silver Pheasant**	**China**

*^1^Novel STs are indicated in bold. ^2^Species isolated campylobacter jejuni in this study are indicated in bold. ^3^Areas in china are indicated in bold. ^4^BNU, Beijing Normal University; BP, Beihai Park; THP, Temple of Heaven Park; CP, Cuihu Park; BWRC, Beijng Wildlife rescue center; BWP, Beijing Wildlife Park; MP, Milu Park; NWP, Niukouyu Wetland Park; BRRC, Beijing Raptor rescue center; WDL, Wild Duck Lake; HL, Hongluo Lake; XR, Xiyu Reservoir.*

### Antibiotic Resistance of *Campylobacter jejuni*

**FIGURE 2:**
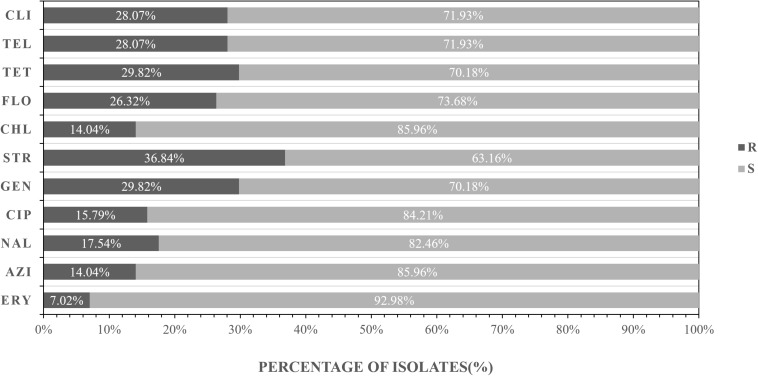
Frequency of resistance to 11 antibiotics among the 57 *Campylobacter jejuni* isolates. R represents resistant, I represents intermediate, S represents sensitive. AZI, azithromycin; NAL, nalidixic acid; CIP, ciprofloxacin; GEN, gentamicin, STR, streptomycin; CHL, streptomycin, FLO, florfenicol; TET, tetracycline, TEL, telithromycin, CLI, clindamycin; ERY, erythromycin.

The antibiotic resistance profile of *C. jejuni* isolates was evaluated by 11 antibiotics according to the recommendations of the Clinical Laboratory Standards Institute (CLSI, 2015). Antibacterial resistance revealed that streptomycin (36.84%) was most common, followed by tetracycline (29.82%), gentamicin (29.82%), clindamycin (28.07%), telithromycin (28.07%), florfenicol (26.32%), nalidixic acid (17.54%), and ciprofloxacin (15.79%), azithromycin (14.04%), chloramphenicol (14.04%), erythromycin (7.02%) (Figure 2 and Table 5).

**TABLE 5:** Prevalence of antibiotic resistance among different species.

	**Aminoglycosides**		**Tetracyclines**	**Lincomides**	**Macrolides Ketolides**			**Quinolones**		**Chloramphenicols**	
						
**Species (Number)**	**GEN**	**STR**	**TET**	**CLI**	**ERY**	**AZI**	**TEL**	**NAL**	**CIP**	**CHL**	**FLO**
Crow (15)	60.00% (9/15)	60.00% (9/15)	26.67% (4/15)	60.00% (9/15)	6.67% (1/15)	26.67% (4/15)	26.67% (14/15)	13.33% (2/15)	20.00% (3/15)	26.67% (4/15)	33.33% (5/15)
Daurian Jackdaw (31)	0.00% (0/31)	6.45% (2/31)	6.45% (2/31)	6.45% (2/31)	0.00% (0/31)	0.00% (0/31)	3.23% (1/31)	9.68% (3/31)	6.45% (2/31)	0.00% (0/31)	6.45% (2/31)
Balck Swan (1)	100.00% (1/1)	100.00% (1/1)	100.00% (1/1)	100.00% (1/1)	0.00% (0/1)	0.00% (0/1)	100.00% (1/1)	0.00% (0/1)	0.00% (0/1)	0.00% (0/1)	100.00% (1/1)
Sliver Pheasant (1)	100.00% (1/1)	100.00% (1/1)	100.00% (1/1)	0.00% (0/1)	0.00% (0/1)	100.00% (1/1)	0.00% (0/1)	0.00% (0/1)	0.00% (0/1)	0.00% (0/1)	100.00% (1/1)
Mallard (6)	50.00% (3/6)	50.00% (3/6)	83.33% (5/6)	50.00% (3/6)	16.67% (1/6)	16.67% (1/6)	50.00% (3/6)	33.33% (2/6)	33.33% (2/6)	33.33% (2/6)	50.00% (3/6)
Mandarin duck (1)	100.00% (1/1)	100.00% (1/1)	100.00% (1/1)	100.00% (1/1)	0.00% (0/1)	0.00% (0/1)	100.00% (1/1)	100.00% (1/1)	0.00% (0/1)	0.00% (0/1)	100.00% (1/1)
Rock Pigeon (2)	100.00% (2/2)	100.00% (2/2)	100.00% (2/2)	100.00% (2/2)	100.00% (2/2)	100.00% (2/2)	100.00% (2/2)	100.00% (2/2)	100.00% (2/2)	100.00% (2/2)	100.00% (2/2)
Total (57)	29.82% (17/57)	36.84% (21/57)	29.82% (17/57)	28.07% (16/57)	7.02% (4/57)	14.04% (8/57)	28.07% (16/57)	17.54% (10/57)	15.79% (9/57)	14.04% (8/57)	26.32% (15/57)

*^1^In parentheses, (A), (B/A), A represent the number of isolates from each bird, B represent the number of isolates from each bird resistance to each antibiotic. ^2^AZI, azithromycin; NAL, nalidixic acid; CIP, ciprofloxacin; GEN, gentamicin; STR, streptomycin; CHL, chloramphenicol; FLO, florfenicol; TET, tetracycline; TEL, telithromycin; CLI, clindamycin; ERY, erythromycin.*

The isolates from different locations and sites showed different antimicrobial profile. In the urban area, the antimicrobial efficiency of three antibiotics commonly used in humans and animals are as follows: streptomycin (62.50%), gentamicin (62.50%), and telithromycin (50.00%) (Table 6). In the suburb area, the rate of antibiotic resistance was low, ranging from 7.32 to 26.83% (Table 6).

**TABLE 6:** Prevalence of antibiotic resistance among different sites.

		**Aminoglycosides**		**Tetracyclines**	**Lincomides**	**Macrolides Ketolides**			**Quinolones**		**Chloramphenicols**	
							
**Location (Number)**	**Site (Number)**	**GEN**	**STR**	**TET**	**CLI**	**ERY**	**AZI**	**TEL**	**NAL**	**CIP**	**CHL**	**FLO**
	BNU (11)	72.73% (8/11)	72.73% (8/11)	36.36% (4/11)	54.55% (6/11)	9.09% (1/11)	27.27% (3/11)	63.64% (7/11)	18.18% (2/11)	27.27% (3/11)	36.36% (4/11)	36.36% (4/11)
Urban (16)	BP (1)	100.00% (1/1)	100.00% (1/1)	100.00% (1/1)	100.00% (1/1)	0.00% (0/1)	0.00% (0/1)	100.00% (1/1)	100.00% (1/1)	0.00% (0/1)	0.00% (0/1)	100.00% (1/1)
	THP (4)	25.00% (1/4)	25.00% (1/4)	25.00% (1/4)	25.00% (1/4)	100.00% (0/4)	25.00% (1/4)	25.00% (1/4)	0.00% (0/4)	0.00% (0/4)	0.00% (0/4)	25.00% (1/4)
	Subtotal (16)	62.50% (10/16)	62.50% (10/16)	37.50% (6/16)	50.00% (8/16)	6.25% (1/16)	25.00% (4/16)	56.25% (9/16)	18.75% (3/16)	18.75% (3/16)	25.00% (4/16)	37.50% (6/16)
	BWRC (4)	50.00% (2/4)	50.00% (2/4)	100.00% (4/4)	50.00% (2/4)	0.00% (0/4)	0.00% (0/4)	50.00% (2/4)	25.00% (1/4)	25.00% (1/4)	0.00% (0/4)	500.00% (2/4)
	CP (25)	4.00% (1/25)	12.00% (3/25)	4.00% (1/25)	4.00% (1/25)	0.00% (0/25)	0.00% (0/25)	4.00% (1/25)	8.00% (2/25)	8.00% (2/25)	4.00% (1/25)	4.00% (1/25)
Suburb (41)	BWP (1)	100.00% (1/1)	100.00% (1/1)	100.00% (1/1)	0.00% (0/1)	0.00% (0/1)	100.00% (1/1)	0.00% (0/1)	0.00% (0/1)	0.00% (0/1)	0.00% (0/1)	100.00% (1/1)
	MP (7)	0.00% (0/7)	14.29% (1/7)	28.57% (2/7)	28.57% (2/7)	0.00% (0/7)	0.00% (0/7)	14.29% (1/7)	14.29% (1/7)	0.00% (0/7)	0.00% (0/7)	28.57% (2/7)
	NWP (4)	75.00% (3/4)	75.00% (3/4)	75.00% (3/4)	75.00% (3/4)	75.00% (3/4)	75.00% (3/4)	75.00% (3/4)	75.00% (3/4)	75.00% (3/4)	75.00% (3/4)	75.00% (3/4)
	Subtotal (41)	17.07% (7/41)	26.83% (11/41)	26.83% (11/41)	19.51% (8/41)	7.32% (3/41)	9.76% (4/41)	17.07% (7/41)	17.07% (7/41)	14.63% (6/41)	9.76% (4/41)	21.95% (9/41)
Beijing (57)	Total (57)	29.82% (17/57)	36.84% (21/57)	29.82% (17/57)	28.07% (16/57)	7.02% (4/57)	14.04% (8/57)	28.07% (16/57)	17.54% (10/57)	15.79% (9/57)	14.04% (8/57)	26.32% (15/57)

*^1^In parentheses, (A), (B/A), A represent the number of isolates from each site, B represent the number of isolates from each site resistence to each antibiotic. ^2^AZI, azithromycin; NAL, nalidixic acid; CIP, ciprofloxacin; GEN, gentamicin; STR, streptomycin; CHL, chloramphenicol; FLO, florfenicol; TET, tetracycline; TEL, telithromycin; CLI, clindamycin; ERY, erythromycin. ^3^ BNU, Beijing Normal University; BP, Beihai Park; THP, Temple of Heaven Park; CP, Cuihu Park; BWRC, Beijng Wildlife rescue center; BWP, Beijing Wildlife Park; MP, Milu Park; NWP, Niukouyu Wetland Park; BRRC, Beijing Raptor rescue center; WDL, Wild Duck Lake; HL, Hongluo Lake; XR, Xiyu Reservoir.*

**FIGURE 3:**
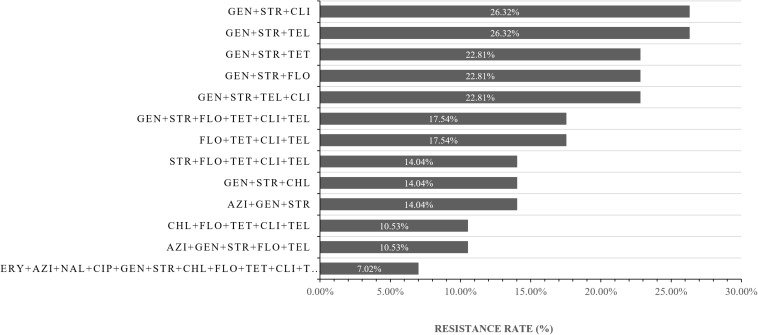
The resistance spectrum of strains of *Campylobacter jejuni* to various antibiotic combinations. The X-axis represents the resistance rate of *Campylobacter jejuni*, The Y-axis represents a series of combination of antibiotics. Thirty-five (61.4%, 35/57) isolates were multi-drug resistance (resistant to more than two antibiotics respectively). AZI, azithromycin; NAL, nalidixic acid; CIP, ciprofloxacin; GEN, gentamicin; STR, streptomycin; CHL, chloramphenicol; FLO, florfenicol; TET, tetracycline; TEL, telithromycin; CLI, clindamycin; ERY, erythromycin.

Multi-drug resistance of bacteria is also common in this study. Nineteen (33.33%, 19/57) isolates were MDR. In detail, The main antibiotics producing drug resistance were streptomycin, gentamicin, and clindamycin (26.32%) (Figure 3 and Supplementary Table S2).

### Antibiotic Resistance Mechanism of *Campylobacter jejuni*

The characteristics of macrolide resistance associated with the genes were analyzed in 4 resistant isolates and 53 susceptible isolates, and this helps investigate the molecular mechanisms of the macrolide-resistant isolates. The A2075G mutation in the 23S rRNA gene, which is responsible for high-level resistance to macrolide, was not detected in all of the resistant strains and susceptible isolates (Supplementary Figure S2A).

All *Campylobacter* isolates were also investigated for the presence of the *tet(O)* gene associated with the resistance to tetracycline. The *tet(O)* marker was detected in all but three resistant strains(3/17) (Supplementary Figure S2B).

To investigate the molecular mechanisms of the fluoroquinolones resistant isolates, Multiplex PCR was designed, which uses three primers in a single reaction. Only the isolates with the gene mutation generated a product with the reverse primer with mutation and the conserved forward primer, whereas all 57 strains generated the gyrA PCR product with reverse and forward conserved primers. 87.50%(7/8) *C. jejuni* resistant isolates generated the specific product in MAMA-PCR that indicated the mutation Thr-86-to-Ile (ACA→ATA for *C. jejuni*), while none of the susceptible strains gave positive results (Supplementary Figure S2C).

All *Campylobacter* isolates were also investigated for the presence of the *aphA-3* gene associated with the resistance to Aminoglycosides. The *aphA-3* marker was detected in all but one resistant strains(1/17) (Supplementary Figure S2B).

### Determination of the Presence of Virulence Genes

To determine whether virulence differences exist among isolates from different wild birds, we tested the virulence genes including the *cdt* gene cluster, *flaA* gene and *cadF* gene, the gene that encodes an adhesin that involves in colonization for all *C. jejuni* isolates.

The results showed that all isolates contained *flaA* genes, *cadF* genes, and *cdt* gene cluster. Further analysis indicated that the truncated *cdt* gene clusters (approximately 1400 bp) only existed in the isolates from crows, daurian jackdaws and silver pheasants. In particular, the truncated *cdt* gene clusters were first detected in *C. jejuni* isolates from silver pheasant. *C. jejuni* isolates from *Corvidae* are more likely to carry truncated *cdt* gene clusters, other species belong to family *Corvids* and *Phasianidae* may also have deletions within the *cdt* Gene Cluster and still need a further survey. These results above indicated that bacteria in the family of *Anatidae* and *Columbidae* might have complete *cdt* gene clusters, but further research is still needed for two families species (Table 7).

**TABLE 7:** Prevalence of CDT isolates from different species.

**Family**	**Species**	**CDT character (%)**
Corvidae	Crow (*Corvus* sp.)	Truncated 100% (15/15)
	Daurian Jackdaw (*Corvus dauurica*)	Truncated 100% (31/31)
Phasianidae	Silver Pheasant (*Lophura nycthemera*)	Truncated 100% (1/1)
Anatidae	Mallard (*Anas platyrhynchos*)	WT 100% (6/6)
	Mandarin duck (*Aix galericulata*)	WT 100% (1/1)
	Black Swan (*Cygnus atratus*)	WT 100% (1/1)
Columbidae	Rock Pigeon (*Columba livia*)	WT 100% (2/2)

*^1^In parentheses, the positive number of isolates from each species of bird.*

## Discussion

*Camphylobacter jejuni* carried by wild birds has been identified to be potentially pathogenic to humans and other animals (Kapperud et al., 2003; Gardner et al., 2012). The majority of studies have focused on food sources that may cause human campylobacterosis, while studies on wild birds are rare, especially in China. In order to get a better understanding of the distribution and transmission of *C. jejuni* in wild birds, we analyzed the prevalence, genetic diversity, antimicrobial resistance and virulence genes of *C. jejuni* in Beijing, China.

In the present study, we found that 10.96% of fecal samples collected from 33 species carried with *C. jejuni*. Previous studies demonstrated that the prevalence of *C. jejuni* among different countries and wild birds species varied from 1.4 to 72.7% (Ramonaite et al., 2015). Among these species we found that the highest prevalence of *C. jejuni* was daurian jackdaw 51.67%, followed by crow 24.19%, silver pheasant 14.29%, black swan 12.5%, mallard 8.57%, rock pigeon 7.69%, and mandarin duck 6.25%. *C. jejuni* isolated from different wild birds with different prevalence which in agreement with other studies that the prevalence of *Campylobacter* spp. in different taxonomic families wild birds were diverse (Waldenstrom et al., 2007; French et al., 2009).

It is believed that the variation of *C.jejuni* prevalence in wild bird species is due to ecological factors including feeding habits, habitat preferences, and migration patterns (Waldenstrom et al., 2002; Colles et al., 2009; Waldenstrom et al., 2010; Colles et al., 2011). Located in urban, BNU had the highest prevalence of *C. jejuni*. Although the proportion of human disease attributable to environmental sources is relatively low, we could not ignore the effect on humans (Wilson et al., 2008; Sheppard et al., 2009; Colles et al., 2011). Most importantly, to our knowledge, it is the first detection of *C. jejuni* carried by black swans in China. These data strengthen the hypothesis that the high prevalence of *C. jejuni* in wild birds might provide evidence of wild birds being a natural reservoir of *C. jejuni* (Oravcova et al., 2014).

Multilocus sequence typing was the golden standard to use for comparison between isolates from different sources because of its high reproducibility and accessible to comparability amongst laboratories worldwide (Dingle et al., 2001). Previous studies have shown that human patients and other animals have the same predominant STs suggesting that these animals are important reservoirs of human domestically acquired infections (Olkkola et al., 2016). From our results, 36 different STs belonging to 4 CCs and unassigned, among which 20 were novel. Of the 20 novel STs, 25% were from one or more new allelic sequences, and a total of 6 novel alleles were found (aspA484, aspA485, glnA673, glyA754, tkt718, tkt719). No new allele sequences were found for another 75% new STs, and these STs resulted from novel combinations of alleles already existed in the PubMLST database. These results indicated that mutation frequency in the MLST alleles is substantially lower than the recombination frequency, which in agreement with previous research (Schouls et al., 2003). ST448, ST951, and ST52 these isolates from daurian jackdaw and black swan, however other researches indicated that all of these three strains isolated from other animals, poultry, and humans (Griekspoor et al., 2010; Olkkola et al., 2016). So wild birds as a potential source of known and novel multilocus sequence types of *C. jejuni* may have the potential to transmit to other animals, poultry, and humans.

The antibiotic resistance profile of *C. jejuni* isolates from these animals was determined using 11 antibiotics. The results of antimicrobial susceptibility testing in this study indicated that the isolates were in general resistant to the tested antibiotics at rates ranging from 7.02 to 36.84%. The high rate of resistance to streptomycin and gentamicin was seen among *C. jejuni* isolates from these birds. All Campylobacter isolates were also investigated for the presence of the *aphA-3* gene associated with the resistance to Aminoglycosides. The most common form of resistance to Aminoglycosides related antibiotics involves the synthesis of 3′-aminoglycoside phosphotransferases [APH(3′)](Gibreel et al., 2004a). The *aphA-3* marker was detected in all but one resistant strains(1/17), which is consistent with previous research results.

From the different locations and sites, the antibiotic resistance profile performed differently. In urban areas, the isolates from wild birds have high antibiotic resistance, which might be due to contaminated environment water. The reasonable interpretation for this difference may be human activities, such as antibiotic abuse, are more active in urban areas than in the suburbs, resulting in birds in different habitats getting different resistant bacteria from the environment. However, the relationship between high antibiotic resistance and contaminated environment water needs further study. As previous study indicated that *C. jejuni* isolates detected in crows and pigeons as potential infection sources to humans (Ramonaite et al., 2015), it was worth noting that four strains from wild birds (crow, mallard, and rock pigeon) are resistant to all 11 antibiotics which may be an important indicator of public health safety.

All wild birds isolates had *flaA* genes, *cadF* genes, and *cdt* gene clusters, which in agreement with the previous study (Sen et al., 2018). The results suggest that these three genes are conserved amongst different sources. As before, *C. jejuni* isolates from crows had a truncated gene cluster of about 1400 bp (Sen et al., 2018), *C. jejuni* isolates from daurian jackdaw also had a truncated gene cluster (Kovanen et al., 2019). However, the most unexpected results were isolates from silver pheasant which also have a truncated gene cluster of about 1400 bp. To our knowledge, it was the first detection of the isolates from silver pheasant also have a truncated gene cluster of about 1400 bp. The CDT toxin is a tripartite protein formed by the expression of three tandem genes, *cdtA*, *cdtB*, and *cdtC* where *cdtB* encodes the active component of the toxin, while *cdtA* and *cdtC* are responsible for binding and internalization of the toxin (Pickett et al., 1996). Some research confirmed that *C. jejuni* 81-176 *cdtBs*^–^ strains were significantly attenuated in HeLa cytotoxicity assays, while still holding some toxigenicity. However, *C. jejuni* NCTC 11168 *cdtB*^–^ strains produced no detectable cytotoxicity in HeLa cell (Purdy et al., 2000). So, if these isolates with a truncated *cdt* gene cluster still retaining the toxigenicity required further verification.

## Conclusion

Wild birds as a reservoir of potentially pathogenic *C. jejuni* strains and can be a vector of disease transmission. However, further studies are needed to link the high occurrence of *Campylobacter* in wild birds to human campylobacteriosis cases and transmission to other animals.

## Experimental Procedures

### Samples Collection and *Campylobacter* Isolation

Five hundred and twenty fecal samples were collected from 33 species in 12 sites, Beijing, China, between January 2018 and April 2018 (Figure 1 and Table 1). Samples were collected in sterile tubes and stored at 4 to 7°C for 2 to 6 h before culturing in Lab (Sen et al., 2018). Fecal samples were inoculated onto Modified Charcoal Cefoperazone Deoxycholate Agar (mCCDA) containing Cefoperazone, Rifampicin, and Amphotericin B (Qingdao Hope Bio-Technology, Co., Ltd.), with incubation at 37°C under microaerophilic conditions (CampyGen; Oxoid Limited, Hampshire, United Kingdom) for 48 to 168 h. Then picked white to translucent colonies subculture onto mCCDA for further characterization.

### *Campylobacter* Identification

All positive samples were used to extract DNA by adding 100 μl 0.25% SDS and boiling in a heater block at 95°C for 10 min, followed by centrifugation at 12,000 *g* for 5 min. Template DNA was stored at −20°C until used for PCR and at least 1 year without any degradation (Sen et al., 2018). Then *Campylobacter* spp. identified by a qPCR method based on the 16S rRNA gene and primers used as previously described (Lund and Madsen, 2006) (Table 8), The qPCR conditions as follows: 1 cycle at 95°C for 10 min, followed by 40 cycles of 95°C for 15 s, 58°C for 30 s, and 72°C for 30 s, with a final cycle of 72°C for 5 min. For determining the presence of *Campylobacter jejuni*, *Campylobacter coli, Campylobacter lari*, and *Campylobacter upsaliensis*, a multiplex PCR method was performed with the isolates using the lipid A gene (*lpxA*) as previously described (Klena et al., 2004), PCR conditions were as follows: 35 cycles of denaturation at 94°C for 1 min, annealing at 50°C for 1 min, extension at 72°C for 1min and a final extension step at 72°C for 5 min. The presence of *flaA*, *cadF* and CDT gene cluster in 57 isolates were determined by PCR using the primer sets described in Table 8 (Nachamkin et al., 1993; Konkel et al., 1999; Bang et al., 2003). The thermocycling conditions which can be found in the respective references in Table 8. The PCR products were detected on 1% Agarose gels and verified by sequencing.

**TABLE 8:** *Campylobacter* specific primers in this study.

**Primer**	**Sequence (5**′**–3**′)	**Gene**	**References**
CampF2	5′-CACGTGCTACAATGGCATAT-3′	*Campylobacter* spp. 16S rRNA	Lund and Madsen, 2006
CampR2	5′-GGCTTCATGCTCTCGAGTT-3′		
Camp P2 (Probe)	**FAM**-5′-CAGAGAACAATCCGAACTGGGACA		
LYA-F	5′-CTTTATGCATGTTCTTCTAAATTT-3′	*cdt* gene cluster	Bang et al., 2003
MII-R:	5′-GTTAAAGGTGGGGTTATAATCATT-3′ (25)		
**Forward**			Klena et al., 2004
lpxA *C. coli*	5′AGA CAA ATA AGA GAG AAT CAG-3′	*C. coli* lpx (391bp)	
lpxA *C. jejuni*	5′ACA ACT TGG TGA CGA TGT TGT A-3′	*C. jejuni* lpx (331 bp)	
lpxA *C. lari*	5′TRC CAA ATG TTA AAA TAG GCG A-3′	*C. lari* lpx (233 bp)	
lpxA *C. upsaliensis*	5′AAG TCG TAT ATT TTC YTA CGC TTG TGTG-3′	*C. upsaliensis* lpx (206 bp)	
**Reverse**			
lpxAARKK2M	5′CAATCATGDGCDATATGASAATAHGCCAT-3′	*cdt* gene cluster	
flaA-F	5′-GGATTTCGTATTAACACAAATGGTGC-3	*fla* (1728 bp)	Nachamkin et al., 1993
flaA-R	5′-CTGTAGTAATCTTAAAACATTTTG-3		
cadFU	5′-TTGAAGGTAATTTAGATATG-3′	*cadF* (400 bp)	Konkel et al., 1999
cadFR	5′-CTAATACCTAAAGTTGAAAC-3′		

### Multilocus Sequence Typing for *Campylobacter jejuni*

Multilocus sequence typing was performed by amplifying and sequencing seven housekeeping genes loci, *aspA, glnA, gltA, glyA, pgm, tkt*, and *uncA*, and primers used for *C. jejuni* are listed in Table 9 (Dingle et al., 2001). The PCR reaction conditions were as follows: initial denaturation at 94°C for 5 min; followed by 35 cycles of 94°C for 2 min, 50°C for 1 min and 72°C for 1 min; last denaturation at 72°C for 7 min. The PCR products were detected on 1% Agarose gels and verified by sequencing. Allele numbers and STs were assigned using the *Campylobacter* MLST database^1^.

**TABLE 9:** Oligonucleotide primers for *Campylobacter* MLST.

	**Dideoxyoligonucleotide primer**			
	
		**Name and sequence**		
			
** Loucs**	**Function**	**Forward**	**Reverse**	**Amplification size (bp)**
asp	Amplification	asp-A9, 5′-AGT ACT AAT GAT GCT TAT CC-3′	asp-A10, 5′-ATT TCA TCA ATT TGT TCT TTG C-3′	899
	Sequencing	asp-S3, 5′-CCA ACT GCA AGA TGC TGT ACC-3′	asp-S6, 5′- TTC ATT TGC GGT AAT ACC ATC-3′	
gln	Amplification	gln-A1, 5′-TAG GAA CTT GGC ATC ATA TTA CC-3′	gln-A2, 5′-TTG GAC GAG CTT CTA CTG GC-3′	1262
	Sequencing	gln-S1, 5′- GCT CAA TTC ATG GAT GGC-3′	gln-S4, 5′- GCA TAC CAT TGC CAT TAT CTC CG-3′	
glt	Amplification	glt-A1, 5′-GGG CTT GAC TTC TAC AGC TAC TTG-3′	glt-A2, 5′-CCA AAT AAA GTT GTC TTG GAC GG-3′	1012
	Sequencing	glt-S3, 5′-CTT ATA TTG ATG GAG AAA ATG G-3′	glt-S8, 5′- TGC TAT ACA GGC ATA AGG ATG-3′	
gly	Amplification	gly-A1, 5′-GAG TTA GAG CGT CAA TGT GAA GG-3′	gly-A2, 5′-AAA CCT CTG GCA GTA AGG GC-3′	816
	Sequencing	gly-S5, 5′- GCT AAT CAA GGT GTT TAT AT-3′	gly-S4, 5′-AGG TGA TTA TCC GTT CCA TCG C-3′	
pgm	Amplification	pgm-A7, 5′-TAC TAA TAA TAT CTT AGT AGG-3′	pgm-A8, 5′-CAC AAC ATT TTT CAT TTC TTT TTC-3′	1150
	Sequencing	pgm-S5, 5′- GGT TTT AGA TGT GGC TCA TG-3′	pgm-S2, 5′- TCC AGA ATA GCG AAA TAA GG-3′	
tkt	Amplification	tkt-A3, 5′-GCA AAC TCA GGA CAC CCA GG-3′	tkt-A6, 5′-AAA GCA TTG TTA ATG GCT GC-3′	1102
	Sequencing	tkt-S5, 5′- GCT TAG ACG ATA TTT TAA GTG-3′	tkt-S6, 5′- AAG CCT GCT TGT TCT TTG GC-3′	
unc	Amplification	unc-A7, 5′-ATG GAC TTA AGA ATA TTA TGG C-3′	unc-A8, 5′-ATA AAT TCC ATC TTC AAA TTC C-3′	1120
	Sequencing	unc-S3, 5′- AAA GTA CAG TGG CAC AAG TGG-3′	unc-S4, 5′- TGC CTC ATC TAA ATC ACT AGC-3′	

### Antimicrobial Susceptibility Testing for *Campylobacter jejuni*

Antimicrobial resistance analysis was performed on 57 *Campylobacter* isolates. All isolates were cultured overnight before testing. The *C. jejuni* strains were tested against phenotypic resistance to 11 antimicrobial agents (erythromycin, azithromycin, nalidixic acid, ciprofloxacin, gentamicin, streptomycin, chloramphenicol, florfenicol, tetracycline, telithromycin, and clindamycin) (Zhongchuan biology technology Company, Qingdao, China) by the agar dilution method according the Clinical and Laboratory Standards Institute (CLSI) guidelines (CLSI, 2015). Mueller-Hinton agar (Oxoid) with dilutions ranging from 64 to 0.5 μg/mL for erythromycin, azithromycin, nalidixic acid, ciprofloxacin, gentamicin, streptomycin, chloramphenicol, florfenicol, tetracycline, telithromycin, and clindamycin was prepared. Subcultured colonies were harvested and suspended in sterile water to a standardized cell density (0.5 McFarland), and 2 μL of approximately 10^4^ CFU of bacteria was pipetted into each well (after diluting in PBS). The plates were incubated under a microaerophilic atmosphere at 42°C for 24 h. The MIC values were defined as the lowest concentration that produces complete inhibition of *C. jejuni* growth. For quality control, the reference strain *C. jejuni* ATCC 33560 was included. The *C. jejuni* isolates were considered resistant to chloramphenicol (CHL), erythromycin (ERY), ciprofloxacin (CIP), nalidixic acid (NAL) tetracycline (TET), streptomycin (STR), and telithromycin (TEL) at MICs of ≥32, ≥ 32, ≥ 4, ≥ 64, ≥ 16, ≥ 16, and ≥ 16 μg/ml, respectively. For gentamicin (GEN), florfenicol (FLO), clindamycin (CLI) and azithromycin (AZI), the isolates with MICs ≥ 8 μg/ml were considered resistant.

### Determination of Mechanisms of Antimicrobial Resistance

All strains tested for the antibiotic resistance were examined for the presence of molecular background of the appearing resistance. For the determination of macrolide resistance, the *23S rRNA* genes mutations were detected by the use of PCR for *C. jejuni* (Zhou et al., 2016). For the determination of fluoroquinolone resistance, the *gyrA* mutations were detected by the use of the Mismatch Amplification Mutation Assay – PCR (MAMA-PCR) suitable for *C. jejuni* (Wieczorek, 2011). The presence of *tet(O)* gene associated with the resistance to tetracyclines was also detected (Wieczorek, 2011). The presence of *aphA-3* gene associated with the resistance to aminoglycosides was also detected (Gibreel et al., 2004a) primers used for determination of Mechanisms of Antimicrobial Resistance are listed in Table 10.

**TABLE 10:** *Campylobacter* specific primers in this study.

**Primer**	**Sequence (5**′**–3**′)	**Gene**	**References**
F1-campy-23S	5′-AAGAGGATGTATAG GGTGTGACG-3′	*23S rRNA*	Zhou et al., 2016
R1-campy-23S	5′-AACGATTTCC AACCGTTCTG-3′		
DMT 1	5′-GGCGTTTTGTTT ATGTGCG-3′	*tet(O)*	Wieczorek, 2011
DMT 2	5′-GTTAAAGGTGGGGTTAT AATCATT-3′		
CampyMAMAgyrA1	5′-TTTTTAGCAA AGATTCTGAT-3′	*gyrA*	Wieczorek, 2011
GZgyrA4	5′-CAGTATAACGCATC GCAGCG-3′		
CampyMAMAgyrA5	5′-CAAAGCATCA TAAACTGCAA-3′		
AphA-3 F	5′-GGGACCACCTATGATG TGGAACG-3′	*AphA-3*	Gibreel et al., 2004a
AphA-3 R	5′-CAGGCTTGATCC CCAGTAAGTC-3′		

The *23S rRNA* genes PCR reaction conditions were as follows: initial denaturation at 94°C for 2 min; followed by 35 cycles of 94°C for 30 s, 55°C for 30 s, and 72°C for 30 s; last denaturation at 72°C for 7 min. The *tet(O)* gene PCR reaction conditions were as follows: initial denaturation at 94°C for 2 min; followed by 35 cycles of 94°C for 30 s, 50°C for 30 s, and 72°C for 30 s; last denaturation at 72°C for 7 min. The *gyrA* gene PCR reaction conditions were as follows: initial denaturation at 94°C for 2 min; followed by 35 cycles of 94°C for 30 s, 54°C for 30 s and 72°C for 30 s; last denaturation at 72°C for 7 min. The *tet(O)* gene PCR reaction conditions were as follows: initial denaturation at 94°C for 2 min; followed by 35 cycles of 94°C for 30 s, 52°C for 30 s and 72°C for 30 s; last denaturation at 72°C for 7 min. The primers used for these three genes are listed in Table 10. The PCR products were detected on 1% Agarose gels and verified by sequencing, and the sequences were analyzed to identify mutations using the BLAST program of the GenBank sequence database.

### Phylogenetic Analysis

The MLST profiles were clustered with the Bionumerics software, version 7.6 by using a categorical coefficient and a graphing method called the minimum spanning tree as described before (Schouls et al., 2004). The minimum spanning tree showing the relatedness of 57 *C. jejuni* strains and other *C. jejuni* strains from the PubMLST database, which was based on the STs. Each ST is represented by a circle that is proportional to the number of isolate species comprising that ST. Circles (STs) are linked by lines indicating allelic variation. The different color of each ST indicates the animal host from which each isolate was recovered (red-human, green -wild bird, blue-chicken, yellow-dog, sky blue-monkey). Thick and short lines connect single-locus variants, thin and longer lines connect double-locus variants and dashed lines represent three or more allele differences. For MLST, a maximum neighbor difference of 2 was used to create complexes. Background shading highlights clonal complexes.

### Accession Number(s)

Multilocus sequence typing sequences of the bird isolates that represented novel STs were deposited in the PubMLST database (see footnote 1) to assign new sequence types and allelic profiles.

### Statistical Analysis

Statistical analysis of the prevalence of positive rate in the *C. jejuni* isolates was performed using the chi-squared test with SPSS version 16.0. A value of *p* < *0.05* was considered to be statistically significant.

## Data Availability Statement

The raw data supporting the conclusions of this manuscript will be made available by the authors, without undue reservation, to any qualified researcher.

## Author Contributions

JD, HH, JL, and CW designed the project. JD and JH performed the main experiments. JD, JL, CW, and ML wrote and revised the manuscript. BJW, BW, HC, JJ, and KS conducted part of the experiments.

## Conflict of Interest

The authors declare that the research was conducted in the absence of any commercial or financial relationships that could be construed as a potential conflict of interest.

## Footnotes

1 http://pubmlst.org/campylobacter/
