# Supplementary material for: Emergence of Genetic Diversity and Multi-Drug Resistant Campylobacter jejuni From Wild Birds in Beijing, China
Source: Front Microbiol. 2019 Oct 29;10:2433. doi: 10.3389/fmicb.2019.02433 (PMC6829156; doi:10.3389/fmicb.2019.02433)
Supplement: Supplementary file 1 [file Table_1.docx]

Table S1 Distribution of 36 STs and allele genes

| **ST** | **CC** | **aspA** | **glnA** | **gltA** | **glyA** | **pgm** | **tkt** | **uncA** |
| --- | --- | --- | --- | --- | --- | --- | --- | --- |
| 52 | ST-52 complex | 9 | 25 | 2 | 10 | 22 | 3 | 6 |
| 448 | U | 61 | 70 | 22 | 18 | 2 | 74 | 47 |
| 692 | ST-692 complex | 37 | 52 | 57 | 26 | 127 | 29 | 23 |
| 951 | U | 18 | 100 | 82 | 24 | 138 | 116 | 63 |
| 953 | U | 18 | 100 | 22 | 104 | 133 | 105 | 6 |
| 991 | ST-692 complex | 37 | 52 | 57 | 26 | 107 | 29 | 23 |
| 995 | U | 2 | 4 | 84 | 105 | 126 | 25 | 57 |
| 999 | U | 64 | 22 | 85 | 142 | 134 | 101 | 16 |
| 1540 | ST-1275 complex | 76 | 33 | 22 | 104 | 43 | 109 | 31 |
| 2367 | U | 2 | 28 | 29 | 28 | 74 | 267 | 124 |
| 3938 | U | 64 | 22 | 68 | 97 | 134 | 301 | 16 |
| 4069 | U | 18 | 2 | 22 | 104 | 1 | 86 | 31 |
| 4382 | U | 258 | 174 | 207 | 193 | 134 | 381 | 284 |
| 4571 | U | 80 | 22 | 72 | 325 | 116 | 423 | 305 |
| 6168 | U | 64 | 22 | 20 | 474 | 134 | 495 | 6 |
| 7805 | U | 64 | 22 | 85 | 98 | 134 | 101 | 16 |
| 9175 | U | 15 | 22 | 78 | 18 | 205 | 101 | 188 |
| 9176 | ST-952complex | 18 | 22 | 22 | 98 | 241 | 381 | 16 |
| 9177 | U | 61 | 70 | 22 | 18 | 43 | 74 | 31 |
| 9178 | U | 64 | 22 | 20 | 98 | 134 | 101 | 16 |
| 9179 | U | 64 | 22 | 85 | 142 | 134 | 17 | 16 |
| 9180 | U | 64 | 226 | 310 | 142 | 134 | 593 | 16 |
| 9181 | U | 64 | 22 | 203 | 100 | 134 | 301 | 16 |
| 9182 | U | 64 | 22 | 310 | 100 | 134 | 54 | 16 |
| 9183 | U | 64 | 22 | 20 | 142 | 134 | 495 | 6 |
| 9184 | U | 64 | 22 | 20 | 100 | 144 | 17 | 16 |
| 9185 | U | 258 | 174 | 31 | 193 | 241 | 160 | 131 |
| 9186 | U | 64 | 22 | 85 | 100 | 134 | 17 | 16 |
| 9190 | U | 37 | **673** | 29 | 64 | 127 | **718** | 23 |
| 9191 | ST-692 complex | 437 | 52 | 57 | 26 | 129 | 29 | 23 |
| 9192 | U | **484** | 33 | 77 | 100 | 885 | 269 | 16 |
| 9194 | U | 64 | 450 | 388 | 100 | 134 | 519 | 16 |
| 9195 | U | 64 | 226 | 85 | 100 | 134 | **719** | 16 |
| 9196 | U | **485** | 430 | 375 | 473 | 885 | 116 | 411 |
| 9197 | U | 18 | 22 | 22 | **754** | 345 | 86 | 47 |
| 9222 | U | 64 | 450 | 203 | 142 | 134 | 593 | 16 |

Note. ^1^U, Unassigned clonal complex. ^2^Novel allele genes are indicated in bold.

Table S2 Antibiotic resistance pattern among different *C.jejuni* strains

|  |  |  | **Macrolides** | | **Quinolones** |  | | **Aminoglycosides Chloramphenicols Tetracyclines lincomides** | | | | | | |
| --- | --- | --- | --- | --- | --- | --- | --- | --- | --- | --- | --- | --- | --- | --- |
| **Number** | **ST** | **CC** | **ERY** | **AZI** | **NAL** | | **CIP** | **GEN** | **STR** | **CHL** | **FLO** | **TET** | **TEL** | **CLI** |
| **1** | **52** | **ST-52 complex** | R | R | R | | R | R | R | R | R | R | R | R |
| **2** | **448** | **U** | R | R | R | | R | R | R | R | R | R | R | R |
| **3** | **448** | **U** | R | R | R | | R | R | R | R | R | R | R | R |
| **4** | **448** | **U** | R | R | R | | R | R | R | R | R | R | R | R |
| **5** | **448** | **U** | S | R | R | | R | R | R | S | R | R | S | R |
| **6** | **692** | **ST-692 complex** | S | S | R | | S | R | R | S | R | R | R | R |
| **7** | **951** | **U** | S | R | S | | S | R | R | S | R | R | R | R |
| **8** | **951** | **U** | S | R | S | | S | R | R | S | R | R | S | S |
| **9** | **953** | **U** | S | S | S | | S | R | R | R | R | R | R | R |
| **10** | **991** | **ST-692 complex** | S | S | S | | S | R | R | R | R | R | R | R |
| **11** | **991** | **ST-692 complex** | S | S | S | | S | R | R | S | R | R | R | R |
| **12** | **995** | **U** | S | S | S | | S | R | R | S | R | R | R | R |
| **13** | **999** | **U** | S | S | S | | S | R | R | R | S | R | R | R |
| **14** | **1540** | **ST-1275 complex** | S | R | S | | S | R | R | R | S | S | R | S |
| **15** | **2367** | **U** | S | S | S | | R | R | R | S | R | S | R | S |
| **16** | **2367** | **U** | S | S | S | | S | R | R | S | S | S | R | R |
| **17** | **3938** | **U** | S | S | S | | S | R | R | S | S | S | R | R |
| **18** | **4069** | **U** | S | S | R | | R | S | S | S | S | R | S | S |
| **19** | **4382** | **U** | S | S | S | | S | S | S | S | S | R | S | S |
| **20** | **4382** | **U** | S | S | S | | S | S | S | S | S | R | S | S |
| **21** | **4571** | **U** | S | S | S | | S | S | S | S | S | R | S | S |
| **22** | **6168** | **U** | S | S | R | | S | S | R | S | R | S | S | R |
| **23** | **7805** | **U** | S | S | R | | R | S | S | S | S | S | S | S |
| **24** | **9175** | **U** | S | S | R | | R | S | S | S | S | S | S | S |
| **25** | **9175** | **U** | S | S | S | | S | S | R | S | R | S | R | R |
| **26** | **9175** | **U** | S | S | S | | S | S | S | S | S | S | S | S |
| **27** | **9175** | **U** | S | S | S | | S | S | S | S | S | S | S | S |
| **28** | **9176** | **ST-952 complex** | S | S | S | | S | S | S | S | S | S | S | S |
| **29** | **9176** | **ST-952 complex** | S | S | S | | S | S | R | S | S | S | S | S |
| **30** | **9177** | **U** | S | S | S | | S | S | S | S | S | S | S | S |
| **31** | **9177** | **U** | S | S | S | | S | S | S | S | S | S | S | S |
| **32** | **9178** | **U** | S | S | S | | S | S | S | S | S | S | S | S |
| **33** | **9178** | **U** | S | S | S | | S | S | S | S | S | S | S | S |
| **34** | **9179** | **U** | S | S | S | | S | S | S | S | S | S | S | S |
| **35** | **9180** | **U** | S | S | S | | S | S | R | S | S | S | S | S |
| **36** | **9180** | **U** | S | S | S | | S | S | S | S | S | S | S | S |
| **37** | **9181** | **U** | S | S | S | | S | S | S | S | S | S | S | S |
| **38** | **9182** | **U** | S | S | S | | S | S | S | S | S | S | S | S |
| **39** | **9183** | **U** | S | S | S | | S | S | S | S | S | S | S | S |
| **40** | **9183** | **U** | S | S | S | | S | S | S | S | S | S | S | S |
| **41** | **9184** | **U** | S | S | S | | S | S | S | S | S | S | S | S |
| **42** | **9185** | **U** | S | S | S | | S | S | S | S | S | S | S | S |
| **43** | **9185** | **U** | S | S | S | | S | S | S | S | S | S | S | S |
| **44** | **9186** | **U** | S | S | S | | S | S | S | S | S | S | S | S |
| **45** | **9190** | **U** | S | S | S | | S | S | S | S | S | S | S | S |
| **46** | **9190** | **U** | S | S | S | | S | S | S | S | S | S | S | S |
| **47** | **9191** | **ST-692 complex** | S | S | S | | S | S | S | S | S | S | S | S |
| **48** | **9192** | **U** | S | S | S | | S | S | S | S | S | S | S | S |
| **49** | **9192** | **U** | S | S | S | | S | S | S | S | S | S | S | S |
| **50** | **9194** | **U** | S | S | S | | S | S | S | S | S | S | S | S |
| **51** | **9195** | **U** | S | S | S | | S | S | S | S | S | S | S | S |
| **52** | **9196** | **U** | S | S | S | | S | S | S | S | S | S | S | S |
| **53** | **9196** | **U** | S | S | S | | S | S | S | S | S | S | S | S |
| **54** | **9197** | **U** | S | S | S | | S | S | S | S | S | S | S | S |
| **55** | **9197** | **U** | S | S | S | | S | S | S | S | S | S | S | S |
| **56** | **9222** | **U** | S | S | S | | S | S | S | S | S | S | S | S |
| **57** | **9222** | **U** | S | S | S | | S | S | S | S | S | S | S | S |

Note. ^1^U, Unassigned clonal complex. ^2^R represents resistant, ^3^S represents sensitive. ^4^Full and abbreviation name of antibiotics were: azithromycin (AZI),

nalidixic acid (NAL), ciprofloxacin (CIP), gentamicin (GEN), streptomycin (STR), chloramphenicol (CHL), florfenicol (FLO), tetracycline (TET), telithromycin (TEL), and clindamycin (CLI),

erythromycin (ERY).


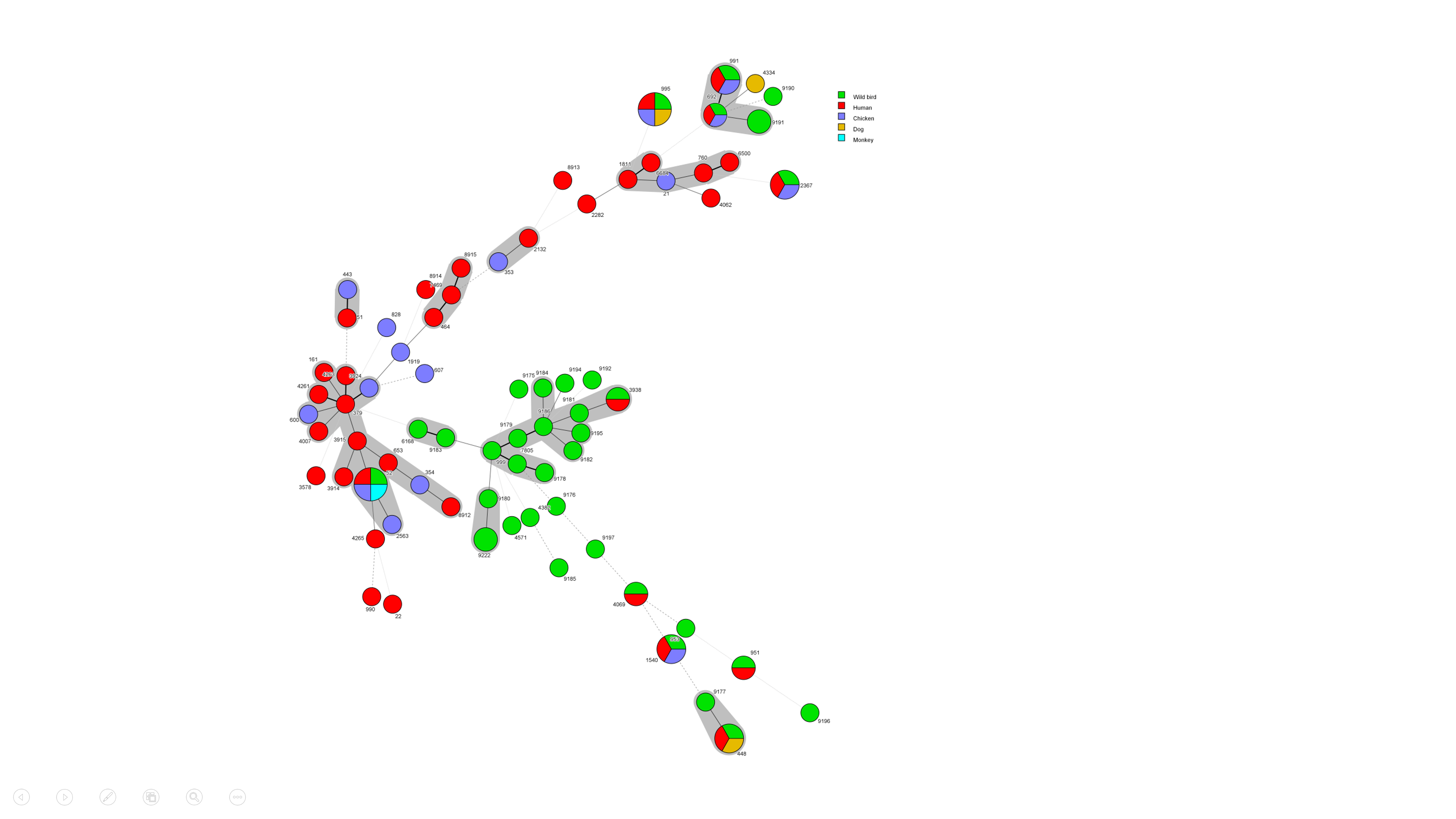


Figure S1| Phylogenetic relatedness of 57 *Campylobacter jejuni* isolates. The minimum spanning tree showing the relatedness of 57 *Campylobacter jejuni* strains and other *Campylobacter jejuni* strains from the pubMLST database which was based on the STs using Bionumerics software, version 7.6. Each ST is represented by a circle; the size of each circle is proportional to the number of isolate species comprising that ST. Circles (STs) are linked by lines indicating allelic variation. Background shading highlights clonal complexes. The colour of each ST indicates the animal host from which each isolate was recovered (red-human, green -wild bird, blue-chicken, yellow-dog, sky blue-monkey). Thick and short lines connect single-locus variants, thin and longer lines connect double-locus variants and dashed lines represent three or more allele differences.


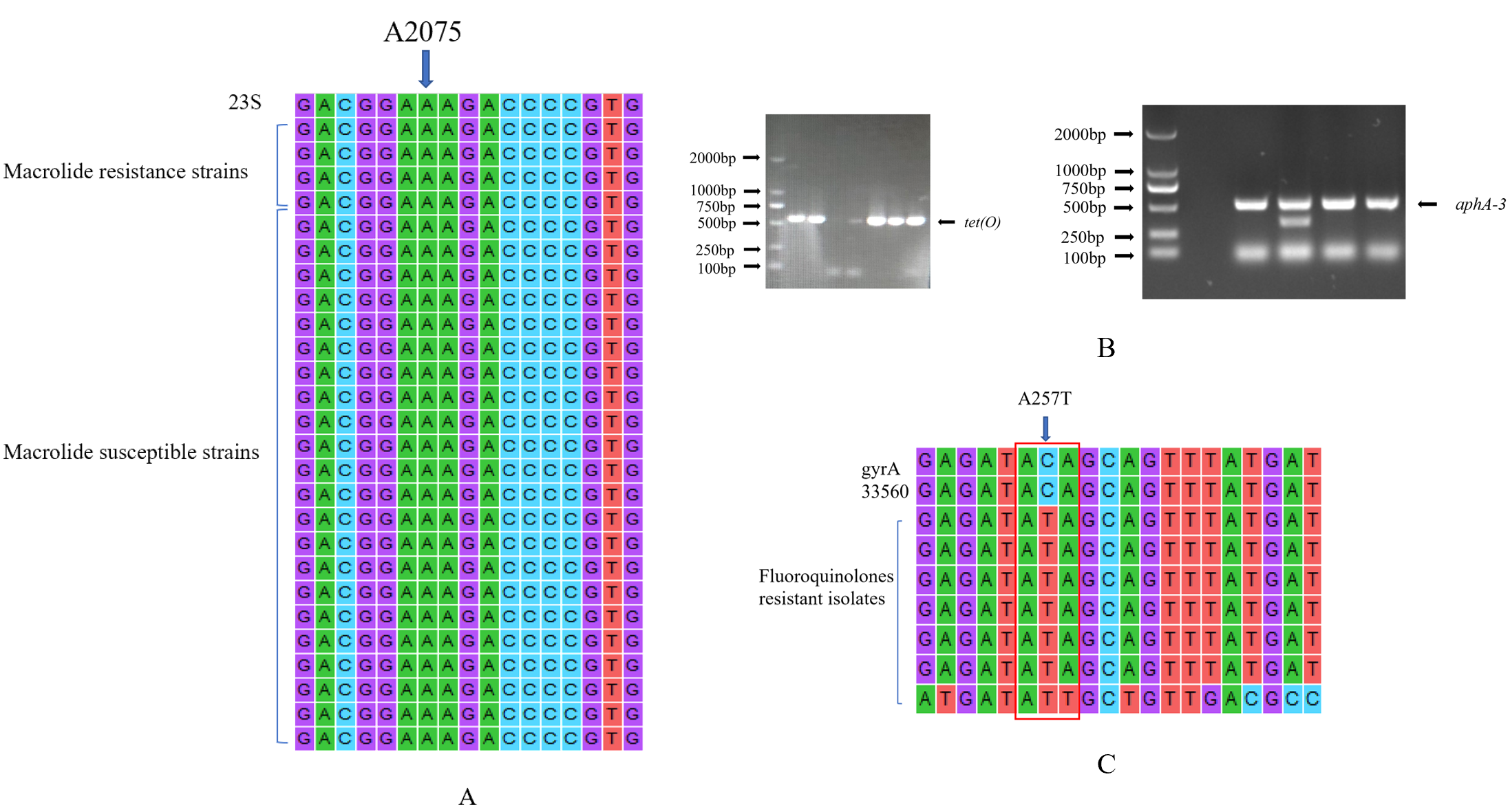


Figure S2 | Antibiotic resistance mechanism of Campylobacter jejuni. A. Nucleotide sequence alignment

of partial gene sequences of *C. jejuni* isolates compared to Campylobacter 23S rRNA sequences from the

GenBank database; B. Agarose gel of detection presence *tet(O)* gene and *aphA-3* gene; C. A Nucleotide sequence alignment

of partial gene sequences of *C. jejuni* isolates compared to Campylobacter *gyrA* sequences from the GenBank

database.
